# A Treatise on Bright’s Disease and Diabetes, with Especial Reference to Pathology and Therapeutics

**Published:** 1905-03

**Authors:** 


### BOOK REVIEWS.

A Treatise on Bright's Disease and Diabetes, with Especial Reference to Pathology and Therapeutics. By James Tyson, M.D., Professor of Medicine in the University of Pennsylvania. Second edition, illustrated. Octavo, 381 pages. Including a section on the Ocular Changes in Bright's Disease and in Diabetes, by G. E. de Schweinitz, Philadelphia: P. Blakiston's Son & Company. 1904. (Price, $4.00.)

Nearly a quarter of a century ago Tyson wrote a book on Bright's disease and diabetes. Although at the time there were many excellent treatises on the subject, this was the work of a master and was received as such. For almost fifteen years prior to the writing of the book Dr. Tyson's work had been largely in the direction suggested by his subject and his rich clinical experience made him one of the authorities of the day.

Since then, very naturally, there has been vast progress in all directions and Bright's disease and diabetes have not been slighted in investigative work, even though the pathologic and etiologic status of diabetes is still in an unsettled state.

Recently Tyson undertook a revision of his book and it is now issued,—a welcome and much-needed addition to the literature of the subjects dealt with. The new work is much broader in scope and much larger than the original, modest masterpiece, and a great deal of valuable material has been added. There are colored plates, original with Tyson, showing the principal forms of Bright's disease, with drawings illustrative of their histology. There are new sections on acute interstitial nephritis, and on the dietetic management of Bright's. The symptomatology of diabetes is clearly set forth; so clearly and in so masterly a manner that one must be inexcusable for error, after even a casual reading of this chapter.

In considering diet in diabetes, there are given analyses of various foods, especially as regards the carbohydrate constituents; and this is particularly valuable, because they are quite independent of the brass-band claims of manufacturers. All of which is interesting and safe to follow.

Another new section of the utmost importance, is that concerning the ocular manifestations of Bright's disease and diabetes, prepared by George E. de Schweinitz and illustrated by colored plates of eye grounds from his own cases.

There is one chapter which will be recognised by those who had the good fortune to read the earlier work. Dr. Tyson has retained almost in its entirety the section on kidney histology.

Aside from the masterly manner in which the subjects are handled, the book is enriched by the presentation of cases from Dr. Tyson's wide experience. These present clinical pictures of vivid type, simple in language, scientifically succinct and unembarrassed by senseless suggestion, or threadbare theory. The book is one of the rarities of latter day medical publication, in that it may be reviewed but not criticised. It is the best work on the subjects now before the profession. N. W. W.

